# Ultra‐Fast and In‐Depth Reconstruction of Transition Metal Fluorides in Electrocatalytic Hydrogen Evolution Processes

**DOI:** 10.1002/advs.202103567

**Published:** 2021-11-12

**Authors:** Pengxia Ji, Ruohan Yu, Pengyan Wang, Xuelei Pan, Huihui Jin, Deyong Zheng, Ding Chen, Jiawei Zhu, Zonghua Pu, Jinsong Wu, Shichun Mu

**Affiliations:** ^1^ State Key Laboratory of Advanced Technology for Materials Synthesis and Processing Wuhan University of Technology Wuhan 430070 P. R. China; ^2^ Foshan Xianhu Laboratory of the Advanced Energy Science and Technology Guangdong Laboratory Xianhu hydrogen Valley Foshan 528200 China; ^3^ Nanostructure Research Centre (NRC) Wuhan University of Technology Wuhan 430070 China; ^4^ Ningxia Key Laboratory of CAE on Intelligent Equipment Ningxia University Yinchuan 750021 China

**Keywords:** density functional theory, hydrogen evolution reaction, in‐depth reconstruction, in situ Raman, transition metal fluorides

## Abstract

Hitherto, there are almost no reports on the complete reconstruction in hydrogen evolution reaction (HER). Herein, the authors develop a new type of reconfigurable fluoride (such as CoF_2_) pre‐catalysts, with ultra‐fast and in‐depth self‐reconstruction, substantially promoting HER activity. By experiments and density functional theory (DFT) calculations, the unique surface structure of fluorides, alkaline electrolyte and bias voltage are identified as key factors for complete reconstruction during HER. The enrichment of F atoms on surface of fluorides provides the feasibility of spontaneous and continuous reconstruction. The alkaline electrolyte triggers rapid F^−^ leaching and supplies an immediate complement of OH^−^ to form amorphous *α*‐Co(OH)_2_ which rapidly transforms into *β*‐Co(OH)_2_. The bias voltage promotes amorphous crystallization and accelerates the reconstruction process. These endow the generation of mono‐component and crystalline *β*‐Co(OH)_2_ with a loose and defective structure, leading to an ultra‐low overpotential of 54 mV at 10 mA cm^−2^ and super long‐term stability exceeding that of Pt/C. Moreover, DFT calculations confirm that F^−^ leaching optimizes hydrogen and water adsorption energies, boosting HER kinetics. Impressively, the self‐reconstruction is also applicable to other non‐noble transition metal fluorides. The work builds the fundamental comprehension of complete self‐reconstruction during HER and provides a new perspective to conceive advanced catalysts.

## Graphical abstract

A new type of ultrafast and completely reconfigurable transition metal fluoride pre‐catalysts, including CoF_2_, NiF_2_, and FeF_3_ (H_2_O)_0.33_, are found during the alkaline hydrogen evolution reaction process. In terms of CoF_2_ serving as a representative, its reconstruction mechanism is uncovered.

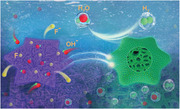

## 1 Introduction

For oxygen evolution reaction (OER) pre‐catalysts in alkaline media, it has been well established that their intrinsic activity originates from the reconstructed intermediates, such as oxides,^[^ ^1^ ^]^ hydroxides,^[^ ^2^ ^]^ and oxy‐hydroxides.^[^ ^3^ ^]^ Unfortunately, for hydrogen evolution reaction (HER) catalysts, to date, only a few studies show that the surface reconstruction can take place and contribute to improving HER performance. Meanwhile, these reports seem to present debatable active sites (after reconstruction), such as reduction to metals^[^ ^2^, ^4^ ^]^ or hydroxylation to hydroxides^[^ ^5^ ^]^ and oxy‐hydroxides.^[^ ^6^ ^]^ Moreover, most of the original catalysts are preserved due to the limited reconstruction degree. Such a complex structure makes it difficult to identify the real origin of activity in the catalysts. Hence, the rational design of rapidly and completely reconfigurable pre‐catalysts during alkaline HER and the revelation of the underlying reconstruction mechanism are of great significance. The key constraint of in‐depth reconstruction is the generation of dense reconstruction layer due to sluggish mass transfer process, which blocks further mass transfer according to Mai et al.^[^ ^7^ ^]^ For deeper or complete reconstruction, the designed pre‐catalysts should possess the advantages of rapid mass transferability and the formation of a loose reconstructible layer. Existing studies found that the introduction of F^−^ makes the surface of catalysts more hydrophilicity,^[^ ^8^ ^]^ thus favoring to fully react with electrolyte to realize preliminary reconstruction. The reconstructible layer presents a highly mesoporous hierarchical structure,^[^ ^9^ ^]^ which enables more electrolyte penetration for deepening the reconstruction. Thus, the introduction of more fluorine into pre‐catalysts probably makes them easier to implement deeper or even complete reconstruction.

Accordingly, a new class of single‐phase target pre‐catalysts, transition metal fluorides, including CoF_2_, NiF_2_, and FeF_3_ (H_2_O)_0.33_, are developed for alkaline HER. As expected, fluorides undergo a successive and rapid self‐reconstruction process owing to the leaching of F^−^ during HER, resulting in great reductions in overpotentials. Especially for CoF_2_, it not only exhibits extraordinary catalytic activity comparable to the benchmark Pt/C but also outputs high stability exceeding Pt/C. By means of in situ Raman together with ex situ X‐ray diffraction (XRD), X‐ray photoelectron spectroscopy (XPS), and spherical aberration correction electron microscopy (AC‐TEM) analyses, we systematically uncover the successive self‐reconstruction process of CoF_2_, in which the mono‐component hexagonal crystalline phase, *β*‐Co(OH)_2_, is presented as about 5 nm nanoparticles (NPs) interconnected into porous and defective interwoven nanosheets (NSs). Also, by combining the density functional theory (DFT) calculations, we further disclose the reconstructive evolution process.

## 2 Results

### 2.1 Synthesis and Structure Characterizations of Pre‐Catalysts

**Figure 1:**
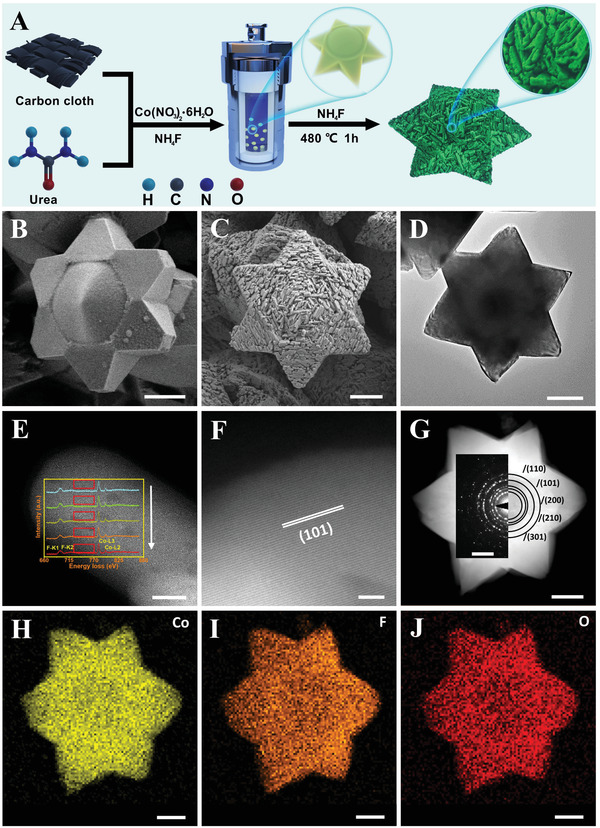
A) Synthetic scheme of hexagram star CoF_2_ supported on CC. B) FESEM image of CoF_1.3_(OH)_0.7_. C) FESEM, D) TEM, E) HAADF‐STEM (inset, EELS spectra integrated from red box region), F) HRTEM, and G) HAADF‐STEM (inset, SAED pattern) images of CoF_2_. H–J) Corresponding EDS elemental mappings. Scale bar: (B, C) 2 µm; (D) 500 nm; (E) 20 nm; (F) 5 nm; (G–J) 200 nm, 5 nm^−1^ (inset).

The synthetic process of CoF_2_ including the growth of precursor and subsequent fluoridation is depicted in **Figure** **1**A. The rosy red precursor with a hexagram star morphology grew on the pre‐cleaned carbon cloth (CC) via a facile hydrothermal strategy (Figure S1A, Supporting Information). From the XRD pattern, it is well‐indexed that the cobalt hydroxide fluoride (CoF_1.3_(OH)_0.7_, JCPDS No. 18–0405) was thoroughly transformed into pink single‐phase cobalt fluoride (CoF_2_, JCPDS No. 71–1969) by reacting with hydrogen fluoride (HF) vapor resulting from the thermal decomposition of NH_4_F (Figures S1B, S2, and S3, Supporting Information). Both oxygen and fluorine signals were detectable by XPS, indicating the adsorbed oxygen species on catalyst surfaces due to exposure to air (Figure S4A, Supporting Information). The relevant peak of adsorbed oxygen species appears at 532.2 eV in the O 1s spectrum (Figure S4B, Supporting Information).^[^ ^10^ ^]^ Meanwhile, the peaks belonging to CoF_2_ locate at 783.0 and 799.1 eV in Co 2p spectrum and 684.8 eV in F 1s spectrum (Figure S4C, D, Supporting Information).^[^ ^8^, ^11^ ^]^ The remaining peaks of 787.8 and 804.7 eV derive from the satellites of Co 2p3/2 and Co 2p1/2.^[^ ^12^ ^]^

From field emission scanning electron microscope (FESEM) images, it is clearly seen that CoF_1.3_(OH)_0.7_ has the shape of a regular hexagram star, with the lateral diameter of about 6 µm and thickness in the range of about 2 to 3 µm (Figure 1B and Figure S5, Supporting Information). After fluorination, the hexagram star structure as a precursor is well preserved and stacked by 3D irregular ribbons with a rough face, which has the high homogeneity of chemical components from edge to center (Figure 1C–E, Figures S6 and S7, Supporting Information). Their lattice fringes with an interplanar distance of approximately 2.64 Å are well indexed to the (101) plane of CoF_2_ (Figure 1F). The rings in the selected area electron diffraction (SAED) pattern of the hexagram star are indexed to the lattice planes of

(110), (101), (200), (210), and (301) (Figure 1G), demonstrating the polycrystalline nature of the prepared CoF_2_. Additionally, the scanning transmission electron microscopy (STEM) element mapping further confirms the homogeneity of CoF_2_ components (Figure 1H–J).

### 2.2 Electrochemical Activity and Stability of HER

#### 2.2.1 Dynamic Activity Variation During HER of Pre‐Catalysts

**Figure 2:**
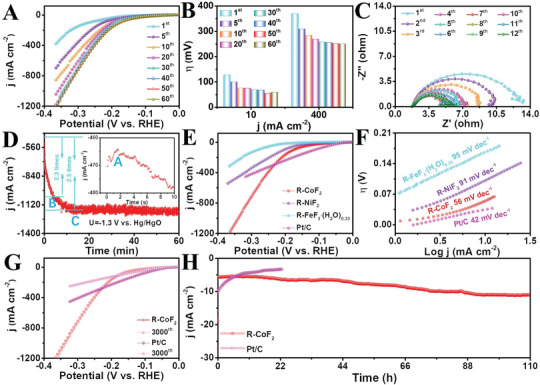
A) Consecutive LSV curves, B) corresponding overpotentials (@10, 400 mA cm^−2^), C) consecutive EIS plots, and D) i‐t curve of CoF_2_. E) LSV curves and F) Tafel slopes of R‐CoF_2_, R‐NiF_2_, and R‐FeF_3_ (H_2_O)_0.33_ in comparison with commercial Pt/C. G) LSV curves of R‐CoF_2_ and Pt/C before and after successive 3000 cycles CV acceleration. H) i‐t curves of R‐CoF_2_ and Pt/C.

To prove that CoF_2_ is capable of speedy realizing reconstruction for promoting catalytic activity during HER, we first tested successive LSV curves under the potentials ranging from −0.8 to −1.3 V versus Hg/HgO in 1 m KOH without iR correction (**Figure** **2**A and Figure S8, Supporting Information). Note that the HER catalytic activity is dynamic with a drastic reduction in overpotential with successive LSV scans, similar to the previous reports on OER,^[^ ^5^, ^13^ ^]^ indicating the possibility of reconstruction of CoF_2_ during HER. When subjected to consecutive LSV scanning for only 50 cycles for the initial CoF_2_ (Figure 2B), the overpotential strikingly decreases by approximately 73 (@10 mA cm^−2^) and 118 mV (@400 mA cm^−2^), resulting in ultra‐low overpotentials of 54 mV at 10 mA cm^−2^ and 251 mV at 400 mA cm^−2^. If further increasing LSV scans, the degree of decrement remains unchanged. The consecutive EIS plots were also recorded at HER occurring. Obviously, a continuous decline in charge transfer resistance (*R*_ct_) is observed with increasing EIS scans, and the final *R*_ct_ of 2.0 Ω following 12 cycles is as low as 36.4% of the initial CoF_2_ value (Figure 2C). As expected, the HER catalytic activity and charge transfer ability of CoF_2_ can be rapidly and significantly enhanced in the course of HER under alkaline electrolyte.

To further observe the dynamic variations, the chronoamperometry (i‐t) curve under a constant potential of −1.3 V versus Hg/HgO was examined. As shown in Figure 2D and Video S1, Supporting Information, its behavior matches well with the LSV changes and involves three key processes driven by the applied potential. Apparently, the current density of the i‐t curve suddenly decreases at the beginning of 2 s. Then, a sharp rise lasts for approximately 5 min. Subsequently, the moderate increase sustains for approximately 10 min and finally keeps nearly at a constant. In contrast, with the initial sample, the obtainable current density is as high as 2.5 times after 15 min of consecutive electrolysis. More importantly, the similarity of dynamic variation during HER is also observed in other non‐noble metal fluorides (NiF_2_ and FeF_3_ (H_2_O)_0.33_) (Figures S9–S12, Supporting Information), which further proves the versatility of transition metal fluoride reconstruction.

#### 2.2.2 Comparison of HER Activity and Stability for Reconstruction‐Derived Components and Pt/C

Additionally, we compared the HER activities of reconstructed fluorides (named as R‐CoF_2_, R‐NiF_2_, and R‐FeF_3_ (H_2_O)_0.33_) supported on CC with commercial Pt/C supported on CC as a benchmark. From Figure 2E, it is obvious that R‐CoF_2_ only needs an ultra‐low overpotential of 54 mV comparable to Pt/C (34 mV) but lower than that of R‐NiF_2_ (101 mV) and R‐FeF_3_ (H_2_O)_0.33_ (159 mV). Its Tafel slope (56 mV dec^−1^) is also closer to that of Pt/C (42 mV dec^−1^, Figure 2F). While the current density exceeding to about 294 mA cm^−2^, the overpotentials of R‐CoF_2_ are lower than those of Pt/C, and its limit current density can be reached up to 1200 mA cm^−2^, indicating a promising application in industrialization. Moreover, there is no degradation for R‐CoF_2_ after the successive CV scans of 3000 cycles and 110 h continuous electrolysis (Figure 2G, H, and Figure S13, Supporting Information). By comparison, the overpotential of Pt/C shifts negatively by about 11 mV at 10 mA cm^−2^, and its current density degrades dramatically with about 68.9% only after 24 h. These demonstrate the reconstruction‐derived component delivers superior catalytic activity and remarkable robustness for HER.

### 2.3 Mechanism Analysis of Reconstruction Processes During HER

#### 2.3.1 In Situ Capture of Structure Evolution Processes

**Figure 3:**
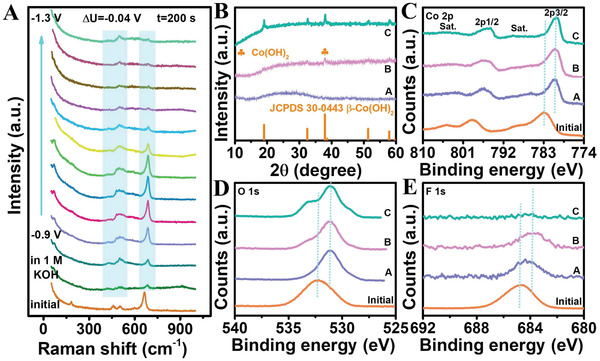
A) In situ Raman spectra of CoF_2_ measured during HER. B) XRD patterns of CoF_2_ at point A–C in the i‐t curve. C) Co 2p, D) O 1s, and E) F 1s spectra of CoF_2_ at point A–C in i‐t curve and initial CoF_2_.

To uncover the dynamic variation of catalytic activity during HER, in situ Raman spectroelectrochemistry was applied for real‐time monitoring of structure information. The signal was captured at the potentials window from −0.9 to –1.3 V versus Hg/HgO in 1 m KOH (**Figure** **3**A and Figure S14, Supporting Information). First, the local structure of fresh CoF_2_ was recorded without the application of potential and electrolyte. It can be seen that the detectable bands centered at 183, 457, 504, and 664 cm^−1^ belong to the Co‐F vibrations in fresh CoF_2_. But all of these bands entirely disappear when soaked in 1 m KOH. Noticeably, three new broad bands of 422, 496, and 685 cm^−1^ originated from the Co—OH vibration in *α*‐Co(OH)_2_ come into being.^[^ ^14^ ^]^ To further corroborate the role of the electrolyte, we dropped the fresh powder sample ink on a transparent glass and soaked it in the electrolyte to observe the color change. Strikingly, the pink CoF_2_ sample turns into blue bluish in a split second, further into brown in a few minutes, and the existence of F^−^ in the electrolyte is supported by ionic chromatography (Video S2 and Figure S15, Supporting Information). This fact demonstrates that the alkaline electrolyte triggers the rapid breakage of Co‐F coordination in CoF_2_ and supplies an immediate complement of hydroxide ions (OH^−^) to form Co—OH coordination in *α*‐Co(OH)_2_, the *α*‐Co(OH)_2_ is unstable and it will fastly transform into *β*‐Co(OH)_2_. While the potentials are applied, the new bands slightly shift to a high wave number, indicating the transformation from *α*‐Co(OH)_2_ to *β*‐Co(OH)_2_. Besides, the bands of *β*‐Co(OH)_2_ become stronger, and the increasing trend is kept until the potential of −1.1 V, confirming the increment of *β*‐Co(OH)_2_. With the potentials further rising, the character bands attributable to *β*‐Co(OH)_2_ are gradually weakened, which could be attributed to the violent production of bubbles at high potentials, affecting the signal capture.^[^ ^14c^ ^]^

#### 2.3.2 Ex Situ Capture of Structure Evolution Processes

Further structure information of species generated during HER was explored by XRD patterns (Figure 3B). We selected three vital points in an i‐t curve as representative of key courses (Figure 2D). At point A, only a broad diffraction peak can be detectable. Combining with the results of in situ Raman, Video S2, and ionic chromatography, Supporting Information, it is deduced that the broad diffraction peak is derived from stable amorphous *β*‐Co(OH)_2_. At point B, a minor crystallization peak assignable to the (101) plane of hexagonal *β*‐Co(OH)_2_ (JCPDS No. 30–0443) phase emerges at 2*θ* = 37.9°. While at point C, apart from the intensity of the above peak increase, four new crystallization peaks corresponding to the (001), (100), (102), and (110) planes of *β*‐Co(OH)_2_ appear in the XRD pattern as well, suggesting bias potential promotes amorphous crystallization.

The chemical states of samples at three vital points and initial CoF_2_ were also investigated. In Figure S16, Supporting Information, the relative intensity of O 1s peaks increases under HER conditions while that of F 1s peaks decreases in comparison with initial CoF_2_. Furthermore, Co 2p and O 1s spectra shift to low binding energies (BEs), yet the shifting degree of BE maintains unchanged after point A (Figure 3C,D). Such peak shifts can be caused by the structural evolution from the Co‐F coordination environment to that of Co—OH.^[^ ^14^, ^15^ ^]^ Although the F 1s spectra display a similar shift trend, the F 1s peak of the sample (point B) further shifts to lower BE and thoroughly vanishes in the sample (point C) (Figure 3E). Gradual shifts and even disappearance of F 1s signal further illustrates that the continuous leaching of F^−^ triggers the successive reconstruction during HER. Besides, the peaks appear at higher BEs and become stronger in the O 1s spectrum, reflecting the adsorbed potassium salts on the catalyst surface.^[^ ^16^ ^]^

#### 2.3.3 Identification of Morphological Evolution Processes

**Figure 4:**
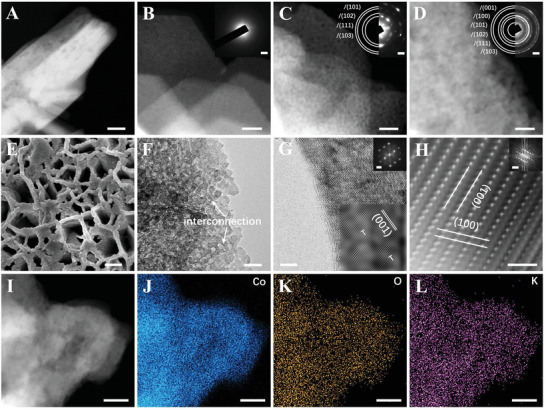
A) HAADF‐STEM image of initial CoF_2_. B–D) HAADF‐STEM images of CoF_2_ at point A–C (inset, SAED pattern). E) FESEM, F) TEM, G) HRTEM (inset, local FFT‐filtered HRTEM image, FFT pattern), and H) atomic‐resolution HAADF‐STEM (inset, FFT pattern) images of CoF_2_ at point C. I–L) HAADF‐STEM image and corresponding EDS elemental mappings of CoF_2_ at point C. Scale bar: (A) 50 nm; (B–D) 10 nm, 2 nm^−1^ (inset); (E) 200 nm; (F) 10 nm; (G) 5 nm, 2 nm^−1^ (inset); (H) 1 nm, 5 nm^−1^ (inset); (I–L) 50 nm.

The aberration‐corrected high angle annular dark‐field scanning transmission electron microscopy (HAADF‐STEM) was conducted to unveil the morphological evolution in HER. Only after 2 s duration under HER test (point A), the polycrystalline ribbon of CoF_2_ is fully converted into an amorphous NS interwoven structure of *β*‐Co(OH)_2_ with plenty of structural defects (**Figure** **4**A,B and Figure S17, Supporting Information). The formation of large surface area and structural defects of NSs favor the contact with more alkaline electrolytes for further reconstruction.^[^ ^17^ ^]^ After continuous electrolysis for 5 min (point B), the amorphous NSs are transformed into low crystallinity NPs of *β*‐Co(OH)_2_. These NPs possess abundant defects and interconnect to form a sponge‐like loose structure (Figure 4C and Figure S18, Supporting Information), allowing more alkaline electrolyte infiltration for catalytic reactions and deeper reconstruction. After complete reconstruction at point C, the catalyst is composed of ultra‐small (≈5 nm) and homogeneous interconnected polycrystalline NPs of *β*‐Co(OH)_2_ with abundant defects (Figure 4D–G and Figure S19A–C, Supporting Information). Furthermore, after complete reconstruction, it inherits the loose and interwoven structure of NSs and the hexagram star frame of CoF_2_, confirmed by the FESEM image (Figure S19D, Supporting Information). Besides, atomic‐resolution HAADF‐STEM image (Figure 4H) elucidates that Co atoms distribute in a layered arrangement, in which the nearest distances between two layers of atoms are 4.64 and 2.73 Å, matching with the (001) and (100) crystal faces of the *β*‐Co(OH)_2_, respectively. STEM‐EDS mappings certify the uniform distribution of reconstruction components and no detectable F signal over the entire region (Figure 4I–L and Figure S20, Supporting Information).

#### 2.3.4 DFT Calculations of Reconstruction Processes

**Figure 5:**
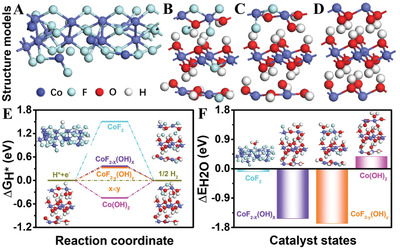
A–D) Optimized structure models, E) Calculated free energy diagram (inset, optimized H* adsorption structure models), and F) H_2_O adsorption energy diagram (inset, optimized H_2_O adsorption structure models) of CoF_2_, CoF_2–x_OH_x_, CoF_2–y_(OH)_y_, and *β*‐Co(OH)_2_.

DFT calculations were further conducted to understand the dynamic reconstruction information of HER, which involves Gibbs free energy of hydrogen adsorption (∆G_H*_) and H_2_O adsorption energy (ΔE_H2O_) of four vital states of CoF_2_, CoF_2–x_(OH)_x_, CoF_2–y_(OH)_y_ (x<y), and *β*‐Co(OH)_2_. Notably, the F atoms of CoF_2_, CoF_2–x_(OH)_x_, and CoF_2–y_(OH)_y_ migrate to the surface after geometry optimization (**Figure** **5**A–C and Figure S21A–C, Supporting Information), which enables them easier to contact electrolytes for achieving spontaneous and continuous reconstruction.^[^ ^8a^ ^]^ For *β*‐Co(OH)_2_, the Co atoms migrate to the surface after geometry optimization (Figure 5D and Figure S21D, Supporting Information). Theoretically, the ∆G_H*_ for ideal catalysts is close to 0 eV, suggesting suitable adsorption and desorption strength of hydrogen. Herein, the computational ∆G_H*_ value of CoF_2_ is 1.51 eV (Figure 5E). Such value is significantly reduced to 0.36 eV with the F^−^ leaching and OH^−^ coordination for CoF_2–x_(OH)_x_, and further decreases with more OH^−^ substituted for F^−^ sites (0.33 eV for CoF_2–y_(OH)_y_). This proves that F^−^ leaching can effectively reduce ∆G_H*_, hence promoting the HER kinetics, which matches well with the results of electrochemical tests. In comparison with CoF_2–y_(OH)_y_, a slight increase in the ǀ∆G_H*_ǀ value is observed for *β*‐Co(OH)_2_ (−0.44 eV). Combining with the morphology and structure analyses of CoF_2–y_(OH)_y_ and *β*‐Co(OH)_2_, we can deduce that the excellent HER catalytic activity of *β*‐Co(OH)_2_ mainly originates from reconstruction‐derived ultra‐small and defective nanostructure. In addition, the H_2_O adsorption energy also shows a similar variation trend (Figure 5F).

## 3 Discussion

The results prove that the obtained target transition metal fluorides are a kind of ideal pre‐catalysts for the realization of ultra‐fast complete self‐reconstruction during alkaline HER. This process depends on three vital factors, namely the unique surface structure of fluorides, alkaline electrolyte, and bias voltage. The fluorides possessing a surface F‐atom enrichment structure with more hydrophilic characters endows the feasibility of continuous self‐reconstruction. When contacting the alkaline electrolyte, the F^−^ is rapidly leached from the fluorides lattice due to high ionicity,^[^ ^8a^ ^]^ and the OH^−^ derived from alkaline electrolyte immediately coordinates with Co sites to form amorphous *α*‐Co(OH)_2_, then rapidly transformed into *β*‐Co(OH)_2_, as confirmed by the previous reports.^[^ ^5^, ^14^, ^18^ ^]^ This preliminary reconstruction brings about striking morphology and structure changes from the dense‐packed irregular ribbons of crystalline CoF_2_ to interwoven NSs of amorphous *β*‐Co(OH)_2_ with abundant structure defects, which enables to contact alkaline electrolyte as much as possible further reconstruction. Moreover, it also contributes to a remarkable reduction in ∆G_H*_, thus providing favorable kinetics for the HER process. As the reaction proceeds, bias potential drives the amorphous crystallization and boosts the process of reconstruction.^[^ ^19^ ^]^ Further F^−^ leaching urges amorphous NSs chalked into interconnected low crystallinity NPs with plenty of defects for deeper reconstruction and causes the preferable HER kinetics. Finally, all the F^−^ ions are leached and the complete reconstruction is realized for pre‐catalysts. The completely reconstructed catalyst is featured by a nanoscale catalytic unit with abundant lattice defects and large specific surface structure, consistent with reported OER pre‐catalysts such as Co_2_(OH)_3_Cl^[^ ^13a^ ^]^ and NiMoO_4_ xH_2_O.^[^ ^20^ ^]^ This unique structure for the reconstruction‐derived mono‐component *β*‐Co(OH)_2_ crystalline phase contributes to the penetration of alkaline electrolyte, exposing more active sites, shortening path charge transfer, thus accelerating the HER process.^[^ ^7^, ^21^ ^]^

## 4 Conclusion

In summary, we construct a new class of transition metal fluoride pre‐catalysts that is capable of achieving ultra‐fast and complete self‐reconstruction, dramatically boosting the HER activity in alkaline media. It only requires an ultra‐low overpotential of 54 mV to deliver the current density of 10 mA cm^−2^ close to commercial Pt/C catalysts, and long‐term stability of 110 h outperforming Pt/C. The experimental results and theoretical calculations co‐confirm the occurrence of the dynamic reconstruction process of fluorides under three vital conditions including the unique surface structure, alkaline electrolyte, and bias voltage. All of them not only accelerate the mass transfer for continuous and deeper reconstruction but also boost kinetics processes of alkaline HER. The reconstruction‐derived mono‐component hydroxides crystalline phase with a nanoscale structure is responsible for the greatly enhanced performance for HER. Our work provides a very important insight for the reconstruction of pre‐catalysts during HER and opens up a novel strategy to design highly efficient catalysts.

## 5 Experimental Section

### Preparation of Pre‐Catalysts

The synthesis process of CoF_2_ was as follows: 1) Co(NO_3_)_2_ 6H_2_O, NH_4_F, and CH_4_N_2_O were successively dissolved in a certain amount of ultrapure water with the molar ratio of 1:4:5 and transferred into reaction kettle after the mixture becomes homogeneous red solution; 2) then added pre‐cleaned CC and mixed into a Teflon‐lined stainless autoclave and the container maintained at 120 °C for 2 h and cooled down to room temperature naturally; 3) the product repeatedly washed with ultrapure water for several times, and finally dried under vacuum at 80 °C for 4 h, the rosy red product loaded on CC were obtained (CoF_1.3_(OH)_0.7_); 4) the obtained CoF_1.3_(OH)_0.7_ and NH_4_F were placed in a tube furnace and annealed at 480 °C for 1 h under an inert atmosphere and cooled down to room temperature naturally; 5) the pink product supported on CC was repeatedly washed with ultrapure water for several times and dried under vacuum for 4 h. Finally, the pink CoF_2_ pre‐catalyst supported on CC was obtained. The synthesis process of NiF_2_ was similar to CoF_2_ just replacing Co(NO_3_)_2_ 6H_2_O with Ni(NO_3_)_2_ 6H_2_O. Because iron fluoride is unstable at high temperature, so FeF_3_ (H_2_O)_0.33_ was obtained at 300 °C for 4 h.

## Conflict of Interest

The authors declare no conflict of interest.

## Author Contributions

P.J., H.J., and S.M. conceived and designed the studies. P.J. synthesized the materials, performed their electrochemical properties, analyzed all the data, and wrote the paper. D.Z. contributed to the materials synthesis and electrochemical measurements. R.Y. and J.W. conducted HAADF‐STEM and HRTEM observations. P.W. performed the DFT calculations, computational models, and result analyses. X.P. and P.J. carried out in situ Raman characterization. D.C., J.Z., Z.P., J.W., and S.M. provided helpful suggestions and revised the manuscript. All authors discussed the results and commented on the manuscript.

## Supporting information

Supporting Information

Supporting Information

Supporting Information

## Data Availability

Research data are not shared.

## References

[1] a) H. Khani , N. S. Grundish , D. O. Wipf , J. B. Goodenough , Adv. Energy Mater. 2019, 10, 1903215;

[2] a) P. W. Menezes , C. Panda , S. Garai , C. Walter , A. Guiet , M. Driess , Angew. Chem., Int. Ed. Engl. 2018, 57, 15237; 30248219 10.1002/anie.201809787

[3] a) Y. Zhu , H.‐C. Chen , C.‐S. Hsu , T.‐S. Lin , C.‐J. Chang , S.‐C. Chang , L.‐D. Tsai , H. M. Chen , ACS Energy Lett. 2019, 4, 987;

[4] Q. Ma , C. Hu , K. Liu , S.‐F. Hung , D. Ou , H. M. Chen , G. Fu , N. Zheng , Nano Energy 2017, 41, 148.

[5] a) C. Karakaya , N. Solati , U. Savacı , E. Keleş , S. Turan , S. Çelebi , S. Kaya , ACS Catal. 2020, 10, 15114;

[6] X. Shang , K. L. Yan , Y. Rao , B. Dong , J. Q. Chi , Y. R. Liu , X. Li , Y. M. Chai , C. G. Liu , Nanoscale 2017, 9, 12353. 28654107 10.1039/c7nr02867a

[7] X. Liu , J. Meng , J. Zhu , M. Huang , B. Wen , R. Guo , L. Mai , Adv. Mater. 2021, 33, 2007344. 10.1002/adma.202007344 34050565

[8] a) P. Chen , T. Zhou , S. Wang , N. Zhang , Y. Tong , H. Ju , W. Chu , C. Wu , Y. Xie , Angew. Chem., Int. Ed. Engl. 2018, 57, 15471; 30216619 10.1002/anie.201809220

[9] B. Zhang , K. Jiang , H. Wang , S. Hu , Nano Lett. 2019, 19, 530. 30517786 10.1021/acs.nanolett.8b04466

[10] C.‐T. Dinh , A. Jain , F. P. G. de Arquer , P. De Luna , J. Li , N. Wang , X. Zheng , J. Cai , B. Z. Gregory , O. Voznyy , B. Zhang , M. Liu , D. Sinton , E. J. Crumlin , E. H. Sargent , Nat. Energy 2018, 4, 107.

[11] S. Wan , J. Qi , W. Zhang , W. Wang , S. Zhang , K. Liu , H. Zheng , J. Sun , S. Wang , R. Cao , Adv. Mater. 2017, 29, 1700286. 10.1002/adma.201700286 28585357

[12] X. Yuan , H. Ge , X. Wang , C. Dong , W. Dong , M. S. Riaz , Z. Xu , J. Zhang , F. Huang , ACS Energy Lett. 2017, 2, 1208.

[13] a) H. Jiang , Q. He , X. Li , X. Su , Y. Zhang , S. Chen , S. Zhang , G. Zhang , J. Jiang , Y. Luo , P. M. Ajayan , L. Song , Adv. Mater. 2019, 31, 1805127; 10.1002/adma.201805127 30633404

[14] a) Z. Liu , H. Liu , X. Gu , L. Feng , Chem. Eng. J. 2020, 397, 125500;

[15] L. Ju , G. Wang , K. Liang , M. Wang , G. E. Sterbinsky , Z. Feng , Y. Yang , Adv. Energy Mater. 2020, 10, 1903333.

[16] P. Ji , H. Jin , H. Xia , X. Luo , J. Zhu , Z. Pu , S. Mu , ACS Appl. Mater. Interfaces 2020, 12, 727. 31841300 10.1021/acsami.9b17960

[17] Y. Wang , Y. Zhu , S. Zhao , S. She , F. Zhang , Y. Chen , T. Williams , T. Gengenbach , L. Zu , H. Mao , W. Zhou , Z. Shao , H. Wang , J. Tang , D. Zhao , C. Selomulya , Matter 2020, 3, 2124.

[18] Y. Zhang , L. Gao , E. J. M. Hensen , J. P. Hofmann , ACS Energy Lett. 2018, 3, 1360. 29911183 10.1021/acsenergylett.8b00514 PMC5996345

[19] J. Liu , Q. Hu , Y. Wang , Z. Yang , X. Fan , L. M. Liu , L. Guo , Proc. Natl. Acad. Sci. U. S. A. 2020, 117, 21906. 32848064 10.1073/pnas.2009180117 PMC7486770

[20] X. Liu , J. Meng , K. Ni , R. Guo , F. Xia , J. Xie , X. Li , B. Wen , P. Wu , M. Li , J. Wu , X. Wu , L. Mai , D. Zhao , Cell Rep. Phys. Sci. 2020, 1, 100241.

[21] a) D. Cao , D. Liu , S. Chen , O. A. Moses , X. Chen , W. Xu , C. Wu , L. Zheng , S. Chu , H. Jiang , C. Wang , B. Ge , X. Wu , J. Zhang , L. Song , Energy Environ. Sci. 2021, 14, 906;

