# Supplementary material for: Ultra‐Fast and In‐Depth Reconstruction of Transition Metal Fluorides in Electrocatalytic Hydrogen Evolution Processes
Source: Adv Sci (Weinh). 2021 Nov 12;9(3):2103567. doi: 10.1002/advs.202103567 (PMC8787395; doi:10.1002/advs.202103567)
Supplement: Supplementary file 1 — Supporting Information [file ADVS-9-2103567-s001.pdf]

WILEY-VCH

© 2020 Wiley-VCH GmbH

ADVANCED SCIENCE
Open Access

Supporting Information

for *Adv. Sci.,* DOI: 10.1002/advs.202103567

Ultra-fast and In-depth Reconstruction of Transition Metal Fluorides in Electrocatalytic Hydrogen Evolution processes

Pengxia Ji, Ruohan Yu, Pengyan Wang, Xuelei Pan, Huihui Jin, Deyong Zheng, Ding Chen, Jiawei Zhu, Zonghua Pu, Jinsong Wu, and Shichun Mu**

Supporting Information

Ultra-fast and In-depth Reconstruction of Transition Metal Fluorides in Electrocatalytic Hydrogen Evolution processes

Pengxia Ji, Ruohan Yu, Pengyan Wang, Xuelei Pan, Huihui Jin, Deyong Zheng, Ding Chen, Jiawei Zhu, Zonghua Pu, Jinsong Wu, * *and Shichun Mu**

P. Ji, R. Yu, P. Wang, X. Pan, H. Jin, D. Chen, J. Zhu, Dr. Z. Pu, Prof. J. Wu, Prof. S. Mu
State Key Laboratory of Advanced Technology for Materials Synthesis and Processing
Wuhan University of Technology
Wuhan 430070, PR China
E-mail: msc@whut.edu.cn

P. Ji, Prof. S. Mu
Foshan Xianhu Laboratory of the Advanced Energy Science and Technology Guangdong Laboratory
Xianhu hydrogen Valley
Foshan 528200, China.

R. Yu, Prof J. Wu
Nanostructure Research Centre (NRC), Wuhan University of Technology, Wuhan 430070, China.
E-mail: wujs@whut.edu.cn

D. Zheng
Ningxia Key Laboratory of CAE on Intelligent Equipment, Ningxia University, Yinchuan 750021, China.

Experimental section

Raw materials

All the reagents were used without further purification, including cobaltous nitrate ($Co(NO_3)_2 \cdot 6H_2O$), nickel nitrate ($Ni(NO_3)_2 \cdot 6H_2O$), ferric nitrate ($Fe(NO_3)_3 \cdot 9H_2O$), ammonium fluoride (NH_4F), urea (CH_4N_2O) and potassium hydroxide (KOH) were purchased from Sinopharm Chemical Reagent Co., Ltd. Nafion (5 wt %), and Pt/C (20 wt %) were purchased from Sigma-Aldrich. The ultrapure water used throughout all the experiments was purified through a Millipore system.

Preparation of working electrode materials

The catalyst suspension ink was prepared by dispersing 5 mg catalyst (Pt/C) in 500 μL of the solution, which is composed of 423 μL isopropyl alcohol, 47 μL ultrapure water, and 30 μL Nafion solution, then followed by ultrasonic dispersion for 30 min. To modify the working electrode, 7 μL of the ink was drop-casted onto a 3 mm carbon cloth and then dried at room temperature naturally to form a catalyst film. The mass loading of all catalysts was about 1 mg cm^{-2}. In addition, fluorides grown on CC were directly served as work electrodes.

Structure characterizations

The constituent phases of precatalysts were recorded via powder X-ray diffraction (XRD, Bruker D8-Advance) using Cu Kα radiation. The microstructures were examined with a field emission scanning electron microscope (FESEM, Zeiss ULTRA-PLUS-43-13), transmission electron microscope (TEM, JEM-2100F), and Thermo Fisher Scientific Titan G80-300 scanning/transmission electron microscopy (STEM). The X-ray energy-dispersive spectrometry (EDS) elemental mapping in the STEM was performed. The surface valence state of the sample was tested by using X-ray photoelectron spectroscopy (XPS, Thermol Scientific Escalab 250Xi, Al Kα radiation). All XPS spectra results were corrected by the C 1s peak at 284.8 eV. Raman spectra and *in-situ* Raman spectra were conducted in 1 M KOH at 25 °C, under testing LSV curves with a scan rate of 0.2 mV s^{-1} between the potential

window of -0.9~-1.3 V vs. Hg/HgO (HORIBA HR EVO, 532 nm). The content of fluoride ion (F^-) by ionic chromatography (DIONEX AQUION).

Electrochemical measurements

The HER electrochemical measurements were performed in a standard three-electrode system on an electrochemical workstation (CHI 660E). To avoid F^- etching glass, we used polytetrafluoroethylene (PTFE) cell for all electrochemical tests. The Hg/HgO and graphite rod were used as the reference electrode and counter electrode, respectively. LSV curves were obtained with a scan rate of 5 mV s^{-1} and a scanning window of -0.8~-1.3 V vs. Hg/HgO in 1 M KOH. The reference electrode was calibrated by a reversible hydrogen electrode (RHE) in advance. All the electrochemical measurements were applied without iR compensation. The electrochemical impedance spectroscopy (EIS) was studied through CHI 660E with the frequency ranging from 10^{-2} to 10^5 Hz at a potential of -1.2 V vs. Hg/HgO. The CV acceleration was conducted between -0.9 and -1.3 V vs. Hg/HgO at a fixed scan rate of 100 mV s^{-1}. A time-dependent current density curve was used to evaluate catalytic activity and stability of HER at -1.3 and -0.98 V vs. Hg/HgO.

Theoretical calculations

DFT calculations were carried out using the CASTEP program on Materials Studio. The Perdew-Burke-Ernzerhof (PBE) functional within the generalized gradient approximation (GGA) was selected to describe the exchange-correlation energy. The projected augmented wave (PAW) method was employed to account for the core-valence interactions and the plane-wave cut-off energy fixed at 300 eV. The Monkhorst-Pack grid k-points of 3×3×1 were selected for the Brillouin zone integration of samples. The vacuum space adopted 20 Å above the surfaces to avoid periodic interactions. The convergence criterions of the energy and force were 1.0×10^{-5} eV/atom and 0.05 eV $Å^{-1}$ for structural optimization. The crystal planes of CoF_2 and $Co(OH)_2$ in DFT calculations are (101) and (001). Apart from atoms on the top layer, all of the other atoms were fixed during the geometry optimizations. Typically, for HER,

the Gibbs free energy (ΔG) of the intermediate H* is obtained based on the equation: $\Delta G_{H*}=\Delta E_{H*}+\Delta E_{ZPE}-T\Delta S$, where ΔE_{H*} represents the binding energy of H species, $\Delta E_{ZPE}-T\Delta S$ represents the change of zero-point energy, and the entropy. Herein, ΔE_{H*} is calculated by the equation: $\Delta E_{H*}=E_{(surf+H*)}-E_{(surf)}-E_{H2}/2$, where $E_{(surf)}$ and E_{H2} represent the energy of bare surface and H_2 gas molecule, respectively. The $E_{(surf+H*)}$ is the energy of the total system with one adsorbed H atom in each unit cell. The value of $\Delta E_{ZPE}-T\Delta S$ is approximately equal to 0.24 eV. Herein, ΔG_{H*} is calculated as: $\Delta G_{H*}= \Delta E_{H*}+0.24$ eV. In addition, water adsorption energies were calculated as follows: $\Delta E_{H2O}=E_{(slab+H2O)}-E_{(slab)}-E_{H2O}$.

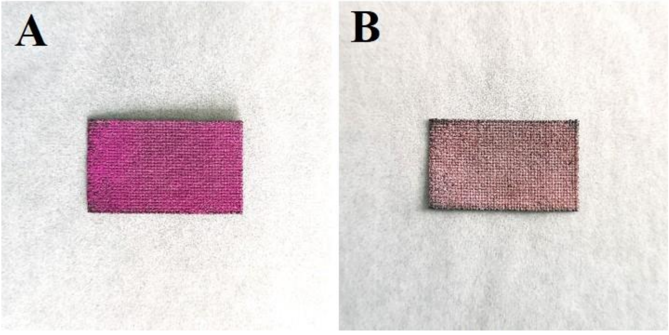

Figure S1. Optical images of (A) $CoF_{1.3}(OH)_{0.7}$ and (B) CoF_2.

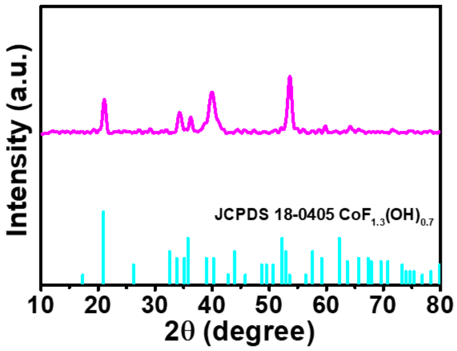

Figure S2. XRD pattern of $CoF_{1.3}(OH)_{0.7}$.

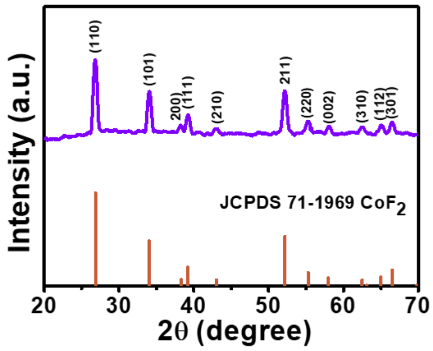

Figure S3. XRD pattern of CoF_2.

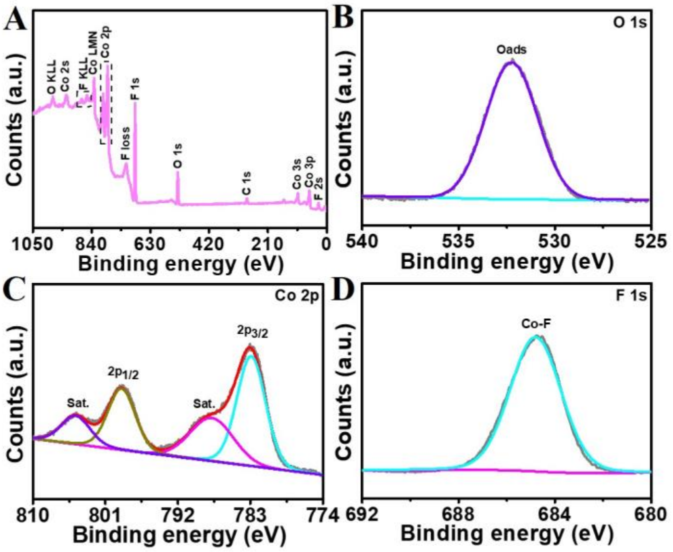

Figure S4. (A) XPS, (B) O 1s, (C) Co 2p and (D) F 1s spectra of CoF_2.

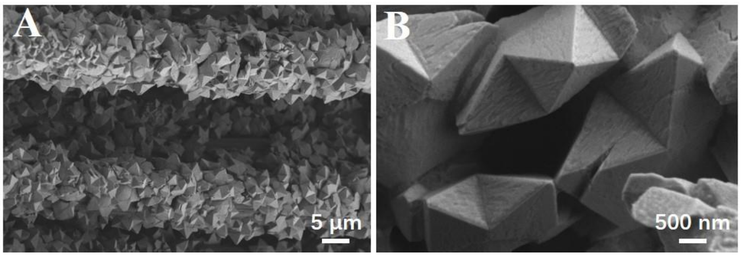

Figure S5. (A, B) FESEM images of $CoF_{1.3}(OH)_{0.7}$.

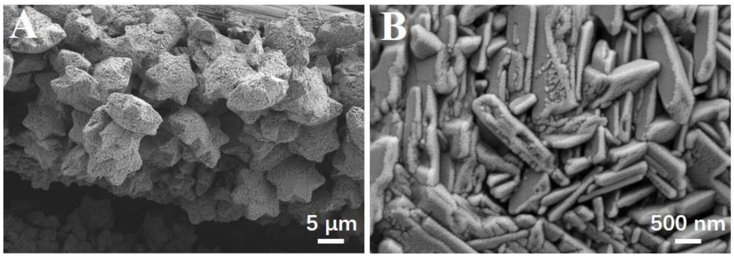

Figure S6. (A, B) FESEM images of CoF_2.

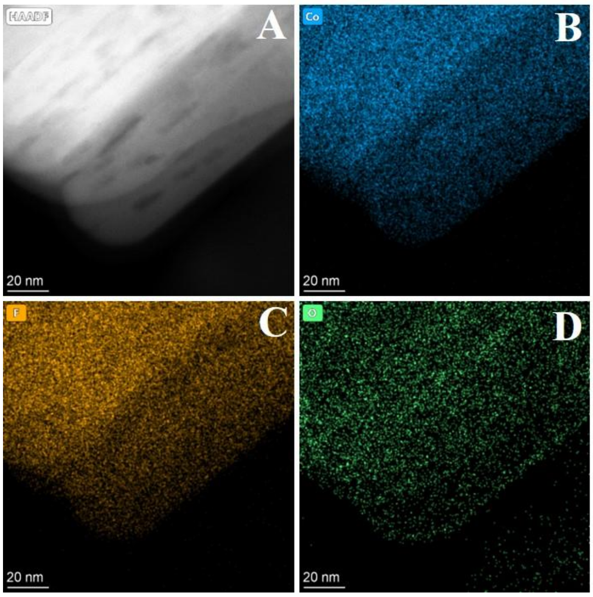

Figure S7. (A) STEM-HAADF image and (B-D) corresponding EDS elemental mappings of CoF_2.

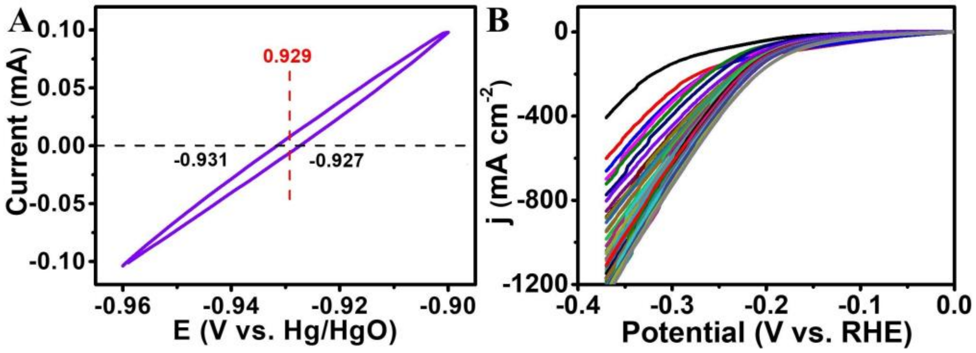

Figure S8. (A) RHE voltage calibration, (B) Consecutive LSV curves of HER for CoF_2.

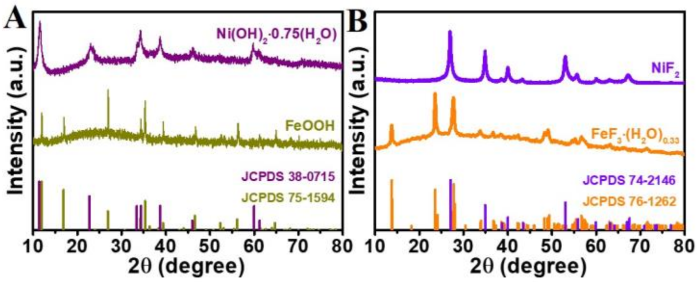

Figure S9. XRD patterns of (A) $Ni(OH)_2 \cdot 0.75H_2O$ and FeOOH,

and (B) NiF_2 and $FeF_3 \cdot (H_2O)_{0.33}$.

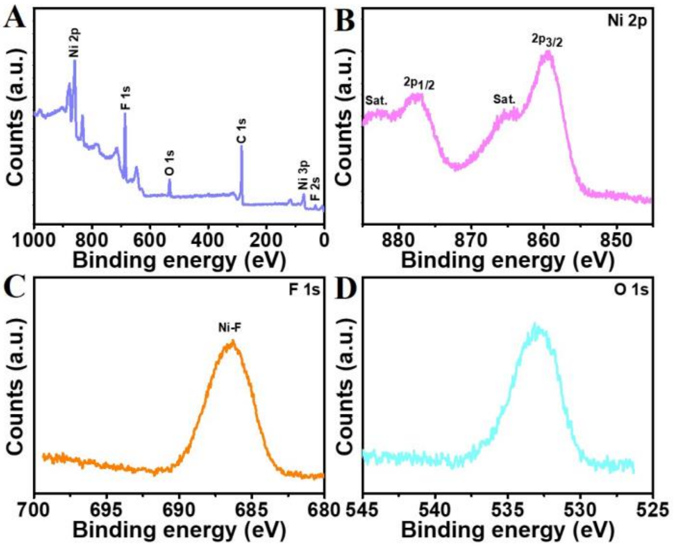

Figure S10. (A) XPS, (B) Ni 2p, (C) F 1s and (D) O 1s spectra of NiF_2.

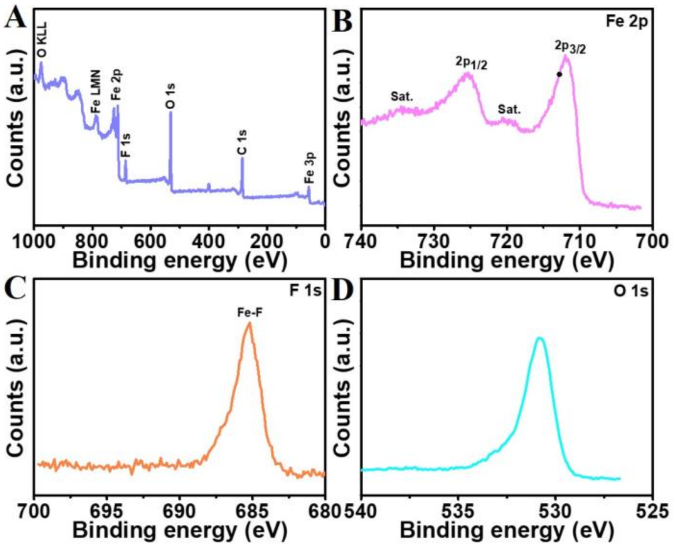

Figure S11. (A) XPS, (B) Fe 2p, (C) F 1s and (D) O 1s spectra of $FeF_3{\cdot}(H_2O)_{0.33}$.

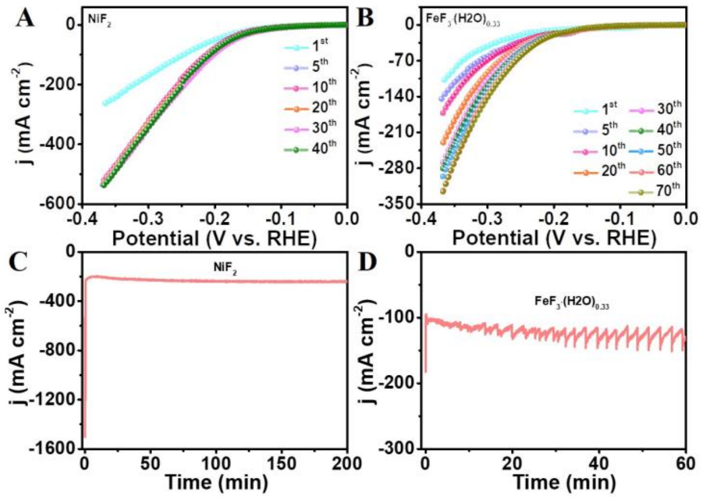

Figure S12. (A, B) Consecutive LSV curves, and

(C, D) i-t curves of NiF_2 and $FeF_3{\cdot}(H_2O)_{0.33}$.

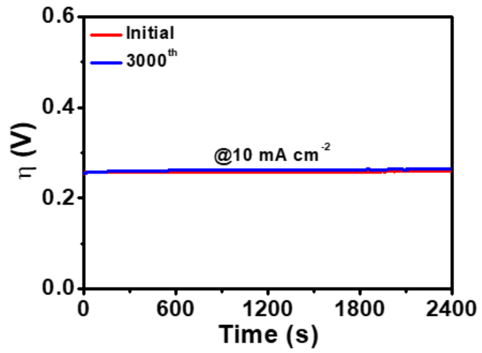

Figure S13. The chronopotentiometry (CP) measurement of R-CoF_2

before and after successive 3000 cycles CV acceleration.

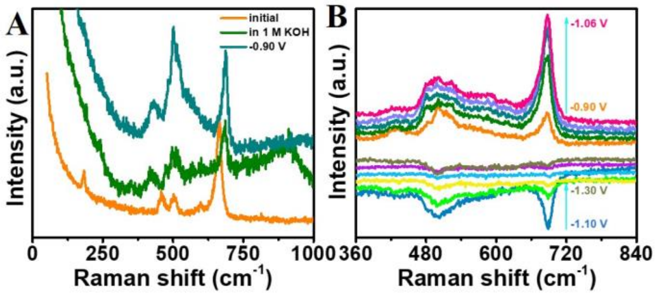

Figure S14. (A, B) *In-situ* Raman of CoF_2.

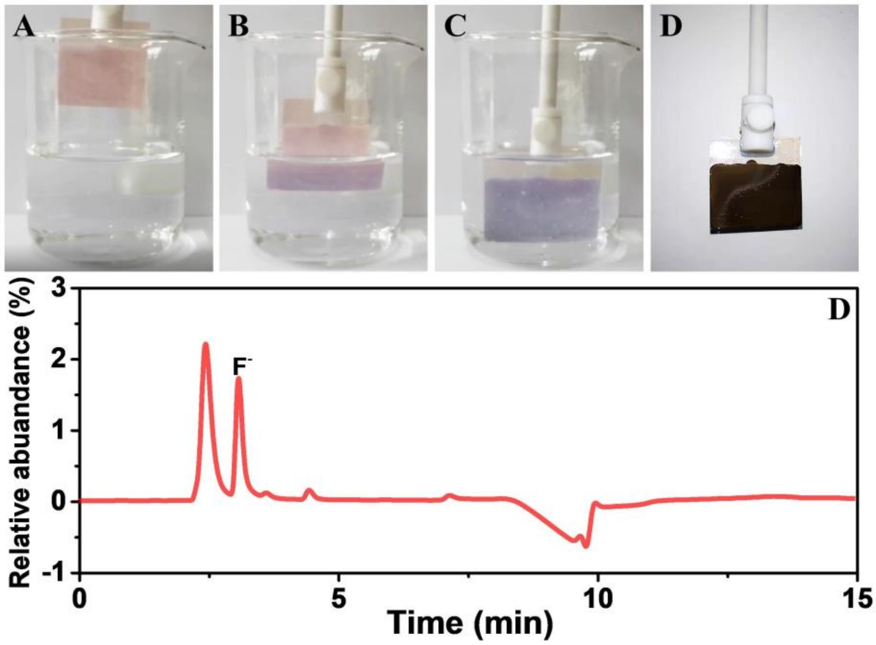

Figure S15. Digital images of the dropped fresh powder CoF_2 on transparent glass (A) before soaked, (B) soaked the half, (C) soaked the all in 1 M KOH, and (D) soaked the all in 1 M KOH for a few minutes, (E) ion chromatogram of electrolyte.

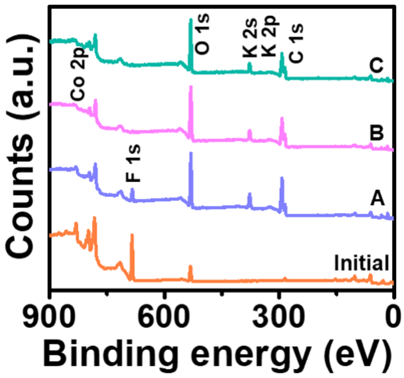

Figure S16. XPS spectrum of CoF_2 at point A-C in i-t curve and initial CoF_2.

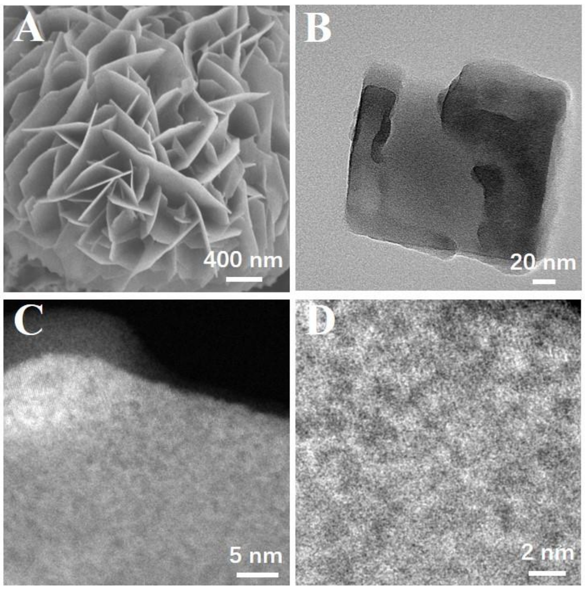

Figure S17. (A) FESEM inamge, (B) TEM image corresponding to inset SAED pattern, and (C, D) HAADF-STEM images of CoF_2 at point A.

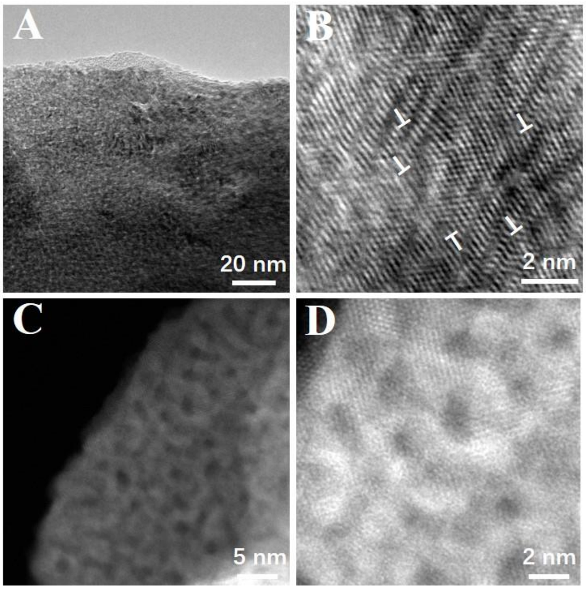

Figure S18. (A) TEM image corresponding to inset SEAD pattern, (B) HRTEM image, and (C, D) HAADF-STEM images of CoF_2 at point B.

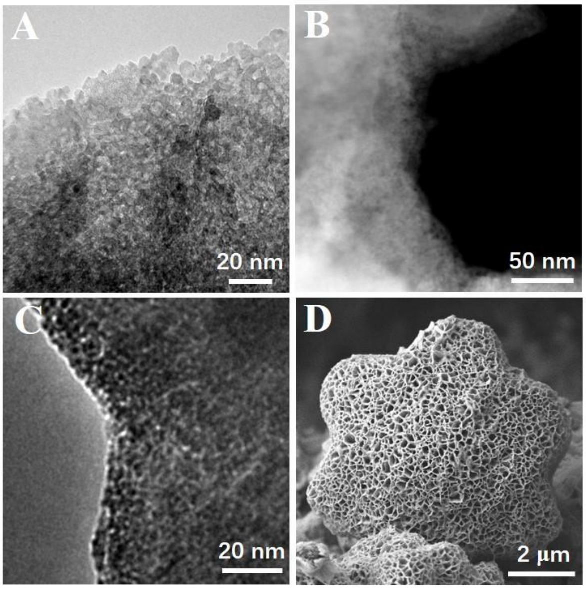

Figure S19. (A) TEM image corresponding to inset SAED pattern, (B, C) HAADF-STEM and (D) FESEM images of CoF_2 at point C.

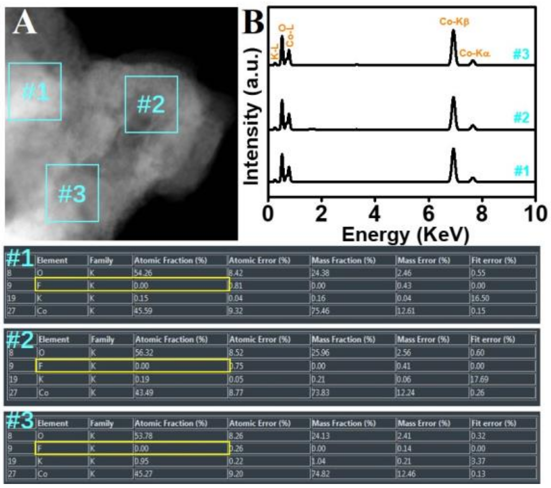

#1

	Element	Family	Atomic Fraction (%)	Atomic Error (%)	Mass Fraction (%)	Mass Error (%)	Fit error (%)
8	O	K	54.26	8.42	24.38	2.46	0.55
9	F	K	0.00	1.81	0.00	0.43	0.00
19	K	K	0.15	0.04	0.16	0.04	16.50
27	Co	K	45.59	9.32	75.46	12.61	0.15

#2

	Element	Family	Atomic Fraction (%)	Atomic Error (%)	Mass Fraction (%)	Mass Error (%)	Fit error (%)
8	O	K	56.32	8.52	25.96	2.56	0.60
9	F	K	0.00	1.75	0.00	0.41	0.00
19	K	K	0.19	0.05	0.21	0.06	17.69
27	Co	K	43.49	8.77	73.83	12.24	0.26

#3

	Element	Family	Atomic Fraction (%)	Atomic Error (%)	Mass Fraction (%)	Mass Error (%)	Fit error (%)
8	O	K	53.78	8.26	24.13	2.41	0.32
9	F	K	0.00	1.26	0.00	0.14	0.00
19	K	K	0.95	0.22	1.04	0.21	3.37
27	Co	K	45.27	9.20	74.82	12.46	0.13

Figure S20. (A) HAADF-STEM image and (B) EDS patterns of CoF_2 at point C.

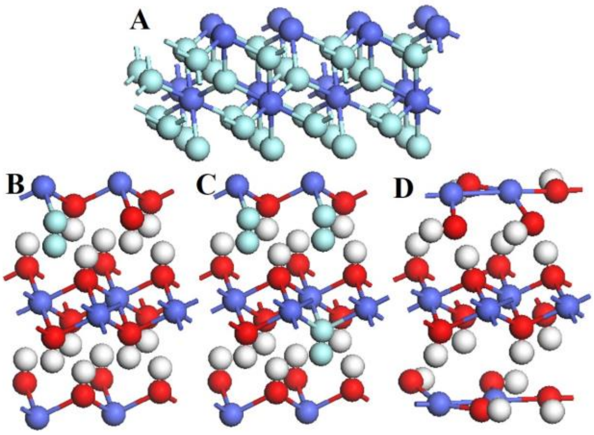

Figure S21. The established theoretical structure model of (A) CoF_2, (B) $CoF_{2-x}OH_x$, (C) $CoF_{2-y}(OH)_y$ and (D) $Co(OH)_2$.
